# Creating opportunities to communicate and connect in a global pandemic: Exploring the experiences of people with aphasia of an online conversation partner scheme

**DOI:** 10.1111/1460-6984.70027

**Published:** 2025-04-01

**Authors:** Naomi de Graff, Lindsey Thiel

**Affiliations:** ^1^ School of Humanities and Social Sciences Leeds Beckett University Leeds UK

**Keywords:** aphasia, conversation partner, communication, technology, online, student

## Abstract

**Background:**

Conversation partner schemes for people with aphasia (PWA) can promote communication and quality of life as well as support skill development for students. Initial evidence indicates online conversation partner formats are acceptable as an alternative to in‐person delivery.

**Aims:**

To evaluate the experiences of PWA of an online conversation partner scheme during the global pandemic.

**Methods & Procedures:**

This qualitative study captured the experiences of eight PWA through semi‐structured interviews. Reflexive thematic analysis was used to analyse the data and to create themes and subthemes, reflecting the experiences of the participants through the interpretation of the researchers.

**Outcomes & Results:**

Two main themes were generated, each with subthemes. The first theme was Communicating and connecting online: ‘It was brilliant’ with subthemes: ‘It help me and I help them’: Mutual benefits, ‘Straight away I managed to speak’: Supported conversations, ‘We got to know each other’: Connecting, and ‘Nine out of ten, easy’: Convenient and easy. The second theme was Being me online: lacks ‘*Je ne sais quoi*’ with subthemes: ‘I like to shake hands’: Missing a human connection, ‘Show me, me self, myself, my broad Yorkshire coming out’: Restricted self‐expression, and ‘Wetherspoons, Wetherspoons’: Loss of the physical environment.

**Conclusions & Implications:**

This study supports the existing evidence that online conversation partner schemes for PWA are successful. It contributes original ideas relating to the acceptability of technology, interaction and self‐expression online for PWA, and considers the benefits of combining both online and face‐to‐face communication and connection opportunities.

**WHAT THIS PAPER ADDS:**

**What is already known on the subject**

- University conversation partner schemes have been shown to improve the communication skills and confidence of PWA and students. A small body of research has shown that online conversation partner schemes are feasible and can be a positive experience for some, but not all, PWA.

**What this paper adds to the existing knowledge**

- Participants found that an online conversation partner scheme during the COVID‐19 pandemic gave them the opportunity to communicate and connect with students, and led to mutual benefits including enjoyment, improved communication skills, confidence and a sense of purpose. They were able to connect through supported conversations and found it convenient and easy to meet online. Despite these benefits, the participants felt that there was something missing in terms of the human connection, self‐expression and the physical environment.

**What are the potential or actual clinical implications of this work?**

- This study suggests that online conversation partner schemes provide an opportunity for those who are restricted in their ability to meet face to face or would prefer the ease and comfort of staying in their own home. However, some people may face more barriers in meeting online due to limited experience, skills or support with technology, and may find it more difficult to communicate, build a connection and express themselves. Either using a combined approach of online and face‐to‐face interactions or providing a choice for PWA will ensure that they are still able to access the psychosocial benefits of participating in a conversation partner scheme.

## INTRODUCTION

Aphasia can have a significant impact on conversations and therefore quality of life (Bullier et al., 2020), given how conversation is central in everyday life (Kagan, 1995, 1998). ‘Supported conversation for adults with aphasia’ (SCA; Kagan, 1998) is an approach to intervention that aims to reduce the psychosocial consequences of aphasia and improve confidence and participation by providing opportunities to have ‘genuine adult conversation and interaction’ (Kagan, 1998: 817). It is based on the concept that interactions are collaborative and the conversational success of people with aphasia (PWA) is dependent on the skill and experience of the person with aphasia, the skill and experience of the conversation partner, and the resources that are available. Kagan (1998) therefore argued that training the conversation partner and making appropriate resources available are as important as directly training the client with aphasia.

Conversation partner training (CPT—also referred to as communication partner training) is an evidence‐based environmental intervention approach that aims to improve the communication, participation and well‐being of PWA. The aim is to train people who interact with a person with aphasia, for example, friends, family and health professionals, to use strategies and resources to support conversations (Simmon‐Mackie et al., 2016). This could include using pictures, maps, gestures, assistive communication devices or written words to support communication, as well as waiting and giving time, checking responses, looking for non‐verbal cues and giving prompts (Cruice et al., 2018). CPT has been shown through systematic reviews to be effective in improving partner skills in supporting communication for people with chronic aphasia (Simmons‐Mackie et al., 2016, 2010). Evidence also shows the value of CPT for PWA as it can increase communicative confidence, identity and well‐being (Simmons‐Mackie et al., 2016) as well as reduce frustration and boredom and increase a sense of social connectedness (Horton et al., 2020). In a recent study which listened to the views of PWA on self‐management, they highlighted the value of conversation partners for practising communication, emotional support and access to technology (Nichol et al., 2022).

### CPT for speech and language therapy students

Speech and language therapy students need to learn how to be a conversation partner for PWA so that they can facilitate conversations with clients and train others to do so. Evidence shows that PWA enjoy and benefit from helping to train speech and language therapy students and being engaged in meaningful activity (Worrall et al., 2011).

Several studies have measured the effects of providing CPT to students both in terms of outcomes for PWA and students. For example, Avent et al. (2009) used reciprocal scaffolding treatment in which the students were trained by a person with aphasia to facilitate conversations. Following the training, the PWA who trained the students made improvements in word fluency, correct information units and type‐token ratio. Finch, Cameron et al. (2017) and Finch, Fleming et al. (2017) conducted a randomized controlled trial to determine the effects of including a lecture for students before they had a conversation with a PWA. The 38 students were randomly allocated to one of two conditions: the full programme, in which they attended a lecture about communication strategies, or only conversation groups, which included getting feedback from PWA. Both groups showed significant improvements in their confidence levels, although significantly more improvements were seen following the full programme. Students received higher competence scores, used significantly more props, and introduced significantly more new ideas than a group that did not attend a lecture. Similarly, in a mixed‐methods design study with nine student participants, Nikkels et al. (2023) evaluated Con‐tAct communication partner training workshops, which included instructional videos and communication skills practice with other students and with two PWA co‐trainers. As well as sharing positive experiences of the training through focus groups, the students showed changes in their communication skills through video analysis, and confidence and knowledge on a self‐report questionnaire.

Other studies have focused on the experiences of those involved through qualitative methods. Jagoe and Roseingrave (2011) thematically analysed reflective letters written by first‐year speech and language therapy students at the beginning and end of a conversation partner scheme. The findings demonstrated that students developed their interpersonal skills and improved their understanding of the social model of disability. McMenamin et al. (2015) used a participatory learning and action (PLA) approach to explore the insider experiences of a conversation partner scheme of five PWA. PWA reported that the programme reduced their negative feelings of communicative incompetence and feelings of marginalization and facilitated communication, reduced exclusion, and led to changes related to their identity, independence and confidence.

### Online CPT for speech and language therapy students

The views of PWA have highlighted the importance of using technology, including to practise communication skills (Nichol et al., 2022). In recent years, a small body of research has explored online student conversation partner schemes. Finch et al. (2020) investigated the feasibility of 33 speech and language therapy students having online conversations with PWA. The students attended a lecture in which they learnt about strategies used to communicate with PWA and had 10‐min conversations online with a PWA 1 week later. Students found the conversations to be positive but challenging. Their self‐rated confidence in communicating with PWA, engaging in an everyday conversation, and obtaining a case history all improved significantly. The authors concluded that delivering the training via telepractice was feasible and valuable for the students.

Similarly, Power et al. (2022, 2020) investigated the efficacy of an online CPT programme for healthcare students. The programme called ‘Communicating with People with Aphasia in Healthcare Contexts’, was based on SCA (Kagan, 1995, 1998), and addressed knowledge of aphasia and its impact on healthcare, facilitative communication techniques for aphasia, and attitudes towards communicating with a person with aphasia. The findings showed that the students improved overall knowledge and attitudes towards aphasia, knowledge of aphasia and knowledge of facilitative communication strategies for engaging with PWA. In their 2020 comparison study, they found no significant difference between online and face‐to‐face groups in relation to knowledge of and attitudes towards aphasia.

Lee et al. (2020) measured the effects and explored the perceptions of five participants with aphasia of an online conversation partner scheme. The PWA took part in a preparatory course conducted by a speech and language therapist. They then practised giving feedback and familiarized themselves with using telepractice equipment. Speech and language therapy students attended a 50‐min lecture about aphasia and communication strategies based on the programme and were trained in communicating effectively via telepractice. The PWA conversed with the speech and language therapy students over 2 days, and after having conversations, the PWA gave the students feedback. Although most ratings of their communication confidence and self‐esteem did not change, there was a significant positive change to their rating of ‘How confident are you about your ability to speak for yourself’. Questionnaire findings showed that all participants with aphasia found it ‘very important’ for speech and language therapy students to have an opportunity to have a conversation and to receive feedback from PWA. Four of the five PWA considered telepractice to be a suitable way to have a conversation with a speech and language therapy student, and they found it a more suitable alternative than via telephone as they could see the student during the interaction. One participant said that he preferred face‐to‐face conversations and experienced technological difficulties. The PWA felt the scheme created new opportunities for students and it enabled them to help students to learn.

### Evaluating online conversation partner schemes during the COVID‐19 pandemic

During the COVID‐19 pandemic in the period 2020–22, people were encouraged to take steps to reduce the spread of the disease, including ‘social distancing’. For PWA, many of whom were already socially isolated due to their communication difficulties, this led to a risk of a further impact on their participation, well‐being and quality of life, as well as reduced connections with family, friends and the community. Despite limited existing evidence to support online approaches, a large amount of university teaching moved online, including conversation partner schemes, to ensure the safety of students and PWA while still providing opportunities for learning, communication and reduced social isolation (Ellis & Jacobs, 2021). Due to the shift in mode of delivery, it was essential to gather evidence to explore the acceptability of online conversation partner schemes for PWA and students.

To date, one published study has evaluated an online conversation partner scheme for students during the COVID‐19 pandemic. Kearns and Cunningham (2023) evaluated the feasibility of an online conversation partner scheme in a university in Ireland during the COVID‐19 pandemic in a mixed‐methods study. The scheme trained students to support conversations with PWA. The students received training sessions and then were paired with PWA to have conversations online. Nineteen students and seven PWA participated in this study. Data collection included a weekly student survey, a post‐CPT questionnaire for students and semi‐structured interviews with PWA. The findings showed that the online CPT was appropriate, although additional online support and training in setting up and familiarization were needed. The PWA found the scheme to be convenient and easy, and the students found the format suitable, although they considered face‐to‐face to be more advantageous. The following themes were found: a shared theme between students and PWA: ‘Technology as a Barrier and Facilitator’, an additional theme from the student data: ‘Conversation Partner Scheme as a Mutually Beneficial Experience and Valuable Learning Experience under the Conditions’, and three additional themes from the interviews with PWA: Valuable Learning Experience, The Power of Conversation and Reflection, and Appraisal and Looking Forward. The authors note that the online format may not be suitable or acceptable to all PWA and students. They concluded that the scheme helped to create the opportunity for communication and reduce social exclusion.

The existing studies have provided useful insight into the outcomes and experiences of PWA and students of online conversation partner schemes, with preliminary evidence. However, there is limited research exploring the perceptions of PWA and students taking part in online conversation partner schemes during COVID‐19. It is vital to understand whether moving to online CPT was an acceptable method of training speech and language therapy students and whether online conversation partner schemes are beneficial for PWA and students.

At Leeds Beckett University, due to the COVID‐19 pandemic, the conversation partner scheme was moved online as it was not possible for students to visit clients in their homes. So they had conversation sessions with clients online through video conferencing platforms. The aim of this project was to evaluate the online conversation partner scheme through exploring the experiences of PWA who took part. This study builds on the work of Kearns and Cunningham (2023) with similar participants and design, but in the context of a university in the North of England. Due to the heterogeneous nature of aphasia and the differences between CPT programmes, further exploration of PWA with different presentations and experiences within another country complements the existing small body of research to support understanding for speech and language therapy programmes about the challenges and benefits of providing this type of online training. Listening to the voices of PWA and their lived experience is essential in guiding CPT and running conversation partner schemes, hence the research question was as follows: What are the experiences of people with aphasia of participating in an online conversation partner scheme?

## METHODS

### Study design

Qualitative research explores the complexity of experiences of individuals and groups of a social phenomenon, through questioning and inductive analysis (Cresswell & Cresswell, 2023). The purpose of this qualitative study was to explore the experiences of PWA participating in an online conversation partner scheme with student speech and language therapists. It is internationally recognized that it is essential to include the views of service users when developing services (Wallace et al., 2024). Through a constructivist paradigm, semi‐structured interviews and reflexive thematic analysis, the researchers aimed to gain an in‐depth understanding of each of the participants’ experiences, hence exploring multiple realities of the experience (Guba & Lincoln, 1994).

Ethical approval for this study was received from Leeds Beckett University Ethics Committee (Ethics approval number 80826). This project was funded by Leeds Beckett University.

### Conversation partner scheme context

The conversation partner scheme at Leeds Beckett University involves a PWA having four, once weekly, supported conversations with two speech and language therapy students in their first year of undergraduate or postgraduate programmes. During the sessions, the PWA and the students engage in conversations about topics of interest and take part in activities such as quizzes or games. Before the scheme started, students attended half‐day CPT to learn about supported conversation skills and supported conversation techniques (Kagan, 1998), and during the sessions, they practised their facilitation skills to support participation in conversations and the various activities. During the COVID‐19 pandemic, students attended the university training session online, during which they were encouraged to think how they could adapt their communication in an online format. PWA were invited to participate in an online format of the scheme.

### Participants

Eight PWA who took part in the online conversation partner scheme volunteered to take part in this study. All participants who were invited agreed to take part in the study. All participants had experienced a stroke and presented with chronic aphasia, ranging from a year to over 20 years post stroke. They had a range of classifications and severities of aphasia. The sample included two females and six males who were all first language English speakers, and their age range varied from being in their 40s to 80s. None of the participants presented with severe auditory impairment or cognitive impairment. They were all able to provide digital written consent in response to accessible, aphasia‐friendly participant information and consent forms. It was explained to the participants that the interview would be made accessible and they could all engage independently in semi‐structured interviews with communication support. Of the eight participants, three had previously taken part in the conversation partner scheme in‐person pre‐pandemic, and five were new to the scheme.

### Procedure

Following the university online CPT, the student peers were paired with a person with aphasia, and they scheduled the video conference meetings at mutually convenient times. Where needed, the students supported the PWA to learn how to access and use the online platform before the sessions commenced. The students and PWA met online once a week for 4 weeks, taking part in supported conversations. After completing the conversation partner sessions, eight PWA took part in semi structured interviews via an online platform. The interviews were carried out by a highly specialized speech and language therapist who was trained and experienced in facilitating conversations with PWA. She used supported conversation techniques throughout the interviews, including use of gestures, visuals and the online platform text function. The data were transcribed from the online platform video recording by a research assistant who was skilled in data transcription and included comments on other methods of communication (e.g., gestures and written words).

### Data analysis

Data analysis was conducted by the two members of the research team, who are speech and language therapy lecturers and researchers. The researchers position themselves subjectively within the research as they have knowledge and experience of teaching students, working with PWA, and running the conversation partner scheme. This subjective insight is viewed as a strength in qualitative research (Braun & Clarke, 2022).

The data were analysed using reflexive thematic analysis, in line with Braun and Clarke's (2013, 2022) guidelines. This entails familiarization, coding of the dataset and searching for initial themes. Themes were then developed and reviewed, leading to defining and naming them. In this case, these were developed collaboratively between the two researchers. Finally, writing up occurred, incorporating analytic narrative supported with data excerpts. To ensure trustworthiness of the analysis, an audit trail was maintained throughout the process with clear documentation of reflexivity, researcher meetings and use of visual thematic mapping (Nowell et al., 2017).

## RESULTS

**FIGURE 1:**
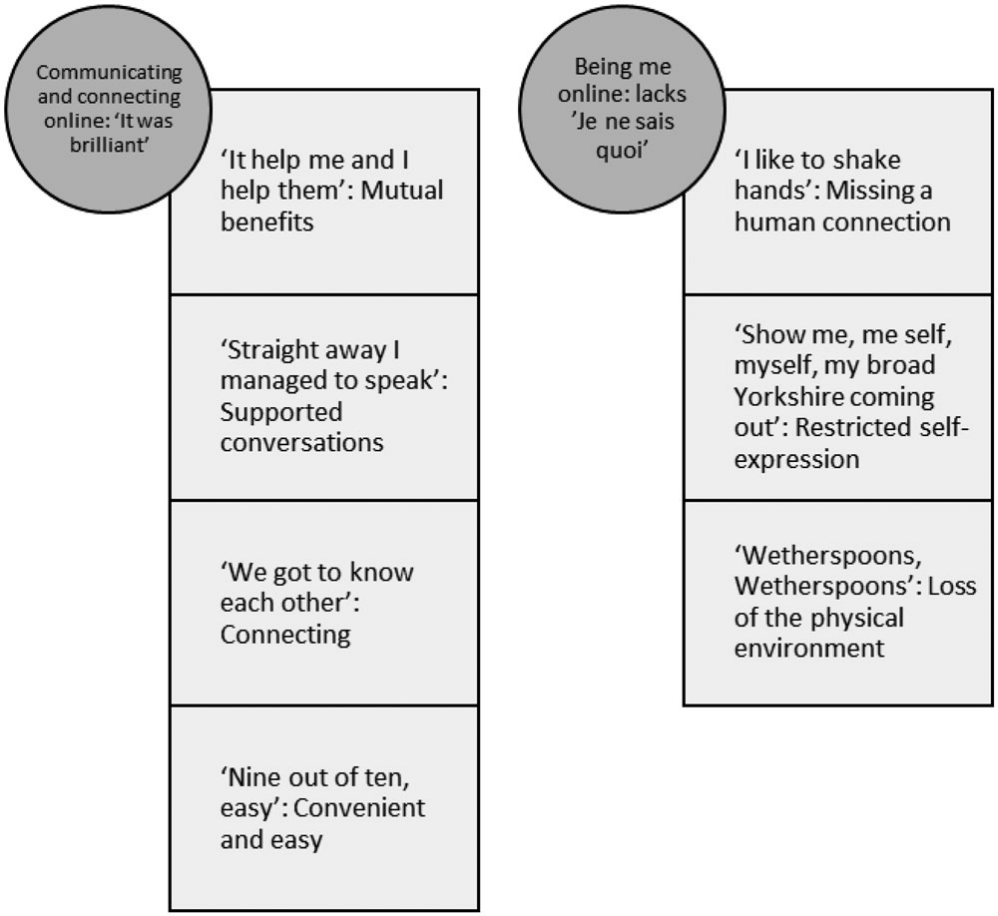
Themes and subthemes.

The following themes and subthemes (Figure 1) were developed collaboratively between the two researchers through reflexive thematic analysis (Braun & Clarke, 2022).

### Theme 1: Communicating and connecting online: ‘It was brilliant’

The first theme captured how much the participants enjoyed the conversation partner scheme and felt that it worked online. There are four subthemes which captured how and why the participants felt that the online conversation partner scheme worked. Beyond simply enjoying it, the PWA felt that there were mutual benefits for themselves and the students (Subtheme 1). All PWA felt that they could still use varied forms of communication and that supported conversations were successful online (Subtheme 2). Participants felt they could make connections and build rapport with the students online (Subtheme 3) and they found the online scheme easy and convenient (Subtheme 4).

#### Subtheme 1: ‘It help me and I help them’: Mutual benefits

The participants recognized the benefits of the online conversation partner scheme both for themselves and the students. First, they expressed how much they enjoyed it. ‘I thought it was first class’ (P2). Taking part during COVID may have increased the anticipation of the sessions with P5 expressing ‘it's erm COVID, lockdown, it was exciting’. P7 shared that he wanted people to talk to ‘Yeah and speak’ adding ‘Yeah and long ago everyone stayed home’. with P5 agreeing that he ‘looked forward to it every week’.

More than simply enjoying it, the participants shared how much they gained from it. For P5 they felt ‘It was good, my my purpose’. P2 shared his views on practising his speech, saying ‘I do like to chat to people and if it helps me to improve my speech skills, that's fantastic’, emphasizing ‘I mean, honestly, it was a real benefit to me’. P1 felt that the conversations helped her feel more confident to explain how others can best support her:

If I not speak I, if I not answer the question I said, ‘you can speak’, I can speak to you but, yeah and also if I not speak very well I said, ‘can you let me know, I can speak again or I can write’.

Participants felt that being part of the conversation partner scheme added to their sense of purpose, as they were also helping the students. P3 proudly said: ‘I've helped them’. They felt that the students were good and developed a range of skills and confidence. ‘The students, the good, very good students’ (P5); ‘They are always helpful’ (P1); and ‘I I could see that they pro process among their their minds’ (P5). They acknowledged that the students were nervous at the start but that this improved over time explained by P7 as ‘But slowly, slowly de de de (gestures happy dancing)’.

P2 reflected further on the scheme allowing them to help the students: ‘I do think it's about getting the experience to help the person to help others’. P2 concluded ‘if it also helps the students to improve their skills that's also, it's a win win situation’.

#### Subtheme 2: ‘Straight away I managed to speak’: Supported conversations

It might be assumed that online communication would be significantly more difficult for people who use a range of communication modalities to convey their message, but the participants felt that they were still able to successfully use a variety of supported conversation techniques. P1 shared ‘I can speak, or I can write’. P3 described using the same communication style online as if the students were at his house, including using gestures, writing, pictures, photos and objects. For this reason, the video was considered to be an important element and P3 stressed that this would not have worked on the phone.

The participants shared that the students contributed to the success of the supported conversations online. When asked if the students prepared resources to talk about P3 replied ‘Oh, loads, loads!’ P2 explained further saying: ‘Yes, yes, yes they did, they did some pictures erm, holidays, pets, family. They were very well prepared for each session’, continuing ‘They did erm a travel erm chat we did a an actual family chat. I took some, I put some photos online from me and it it time just flew by’.

The students also facilitated communication. P6 gave a thumbs up and a ‘10’ to the students giving him enough time to communicate. P7 described the students using cueing ‘teacher “hi” and chatting chatting and stop oh (points to head to suggest difficulties word finding) and dog, dog, dog, and student, the “DOG” and tiny, tiny (gesture to mouth)’ and P5 discussed them slowing down for him: ‘Sorry, have to slow down all the time, slow down, its more for myself. The chattering its its my mind lost’.

An additional benefit, relating to COVID‐19, made communicating online easier. P7 said that it was easier as people were not wearing masks. Online he was able to see people's faces, and they could see his. He could go slowly and pronounce his words to others more clearly for them to understand him: ‘Me alright … fine but slowly, slowly, mouth, mouth (gestures pronouncing words slowly)’.

P5 reflected on communicating online in relation to ending conversations, saying ‘Almost easier, almost easier. … Yeah, I can get up and walk away. I switch you your yourself off, that's it, isn't it?’

#### Subtheme 3: ‘We got to know each other’: Connecting

PWA also felt that the scheme was successful as they were able to connect with the students and build a relationship with them online. P4 rated this ‘10’, the same as if the students were in the room with him, saying he felt ‘comfortable’ with the students. P5 reflected that as they got to know each other, conversation flowed: ‘You know the students and we have freely asked questions, little grins and seriousness, yes. The students err animated’. P2 put it, ‘And I found the experience extremely useful and I can only say that my take on them was that it worked very well. I can't tell you how useful it was I mean, the hour that we had passed very very quickly and we found so much to talk about’.

Participants felt that their ability to connect online with the students was also influenced by their own personalities. P2 described himself as ‘a very determined guy’ and ‘a people person’. P5 felt embarrassed when first meeting the students as he felt like an ‘old fogey’ but soon felt able to connect with them as he saw himself as someone who likes to try new things and ‘not a shrinking violet at all’.

#### Subtheme 4: ‘Nine out of ten, easy’: Convenient and easy

The participants all agreed that another reason the scheme worked was due to the convenience and ease of being online. P7 shared: ‘house morning easy. “Hi” oh and yeah “hi”, yeah fine … and dog, dog “hi.”’ P1 found it more convenient than when she previously had to wait for students to travel to their home: ‘Face to face and they are, for example, if we, if they're going to, if they were going here from … we had to wait’. P4 scored being online 10, saying ‘that's how I prefer it’, which he explained was because: ‘Well, yes because I don't dribble, travel’. P5 emphasized being online was ‘one hundred percent better’ and discussed the many benefits, including efficiency, not having to travel, not burning fuel, and not ‘half a day lost’ or spending time in ‘dreary dreary rooms’. P8 shared that she would not have been able to take part in the scheme in person due to where she lives so being online meant she could be involved.

The participants expressed that using technology was also convenient and easy and did not cause any problems, with P2 commenting ‘perfect, perfect’. Not all the participants were technology users: P5 said ‘I find myself not a student of technology at all’ but had no problems. Other participants were used to using video conferencing. P4 explained that he had been having conversations online for a year already: ‘Yeah, a, we've been talking at least one year’ and P7 used them for work: ‘And me, long ago, computer’ … ‘Yeah, and me hundreds, hundreds, “morning, hi yeah fine” yeah, chatting, chatting, chatting. Me, fine’. P2 also shared: ‘I've always used computers. I didn't learn computers when I first started but I soon found out about them. And I'm not a techy but I'm quite comfortable with them’.

All the participants reported that they did not experience any significant technology problems, although they did acknowledge that they could: ‘never rely completely with technology I things happen and you've always got to solve it …’ (P2). One reason for not feeling concerned with the technology was, for some participants, that family members were there to help with any issues. P1 joked ‘I think, I dunno, my PA, my husband (laughing) had to, what is the words. If he, if the computer is broken, my husband has to mend it or phone some up something, yep’.

### Theme 2: Being me online: lacks ‘*Je ne sais quoi*’

The second theme recognized that despite the overall success of the online conversation partner scheme, it lacked something that was hard to pinpoint and define. The participants felt it related to lacking in three possible areas: human connection (subtheme 1), not being able to fully express themselves and their personalities (subtheme 2) and loss of the same physical environment (subtheme 3).

#### Subtheme 1: ‘I like to shake hands’: Missing a human connection

All the participants felt the online conversation partner scheme was successful, but some acknowledged that it was not the same as meeting people in person, as P5 put it: ‘It's better than nothing at all but it's dividing, a little dividing. Ninety‐five or percent alright’. P1 felt that when she was with someone in person it meant ‘I can smile, and I can can write and have coffee or or something like that’. Some of the participants felt that this related to their age and their appreciation of traditional interactions:

Yes, a bit, old fashioned way. I'm sorry but students, they take it all in quickly. People erm pensioners more but more but set on, set your ways, have you set my ways and in seventy plus, set in my ways a lot. Can't express myself, can't focus, I know what I want to say but can't say it. … Yes shaking hands, you can't shake your hands. Greetings because erm sorry, smell no no, it's not smells at all, it's the screen is this the screen is a barrier which is not too much a barrier but almost ah just a barrier. (P5)

Given that being online was successful but lacked something, the participants felt a balance of online and face to face would be a solution, as P5 said: ‘Maybe one visit face to face then Zoom that'll do or Teams’. P2 felt it is important to: ‘find the correct balance of people and I think it's a marriage between virtual and actual’. P2 went on to summarize:

It's *je ne sais quoi* I think is the French expression, I don't know what it is but I do feel that you've got to, it's probably more relaxed you've not got to set it up quite the same way. And if it's the only way to do it, is virtual, it's a good system, but of course you can intersperse some face to face actual, that gets the best of both worlds.

#### Subtheme 2: ‘Show me, me self, myself, my broad Yorkshire coming out’: Restricted self‐expression

All the participants agreed that supported conversations were successful online but felt that something was lacking in their ability to express themselves in conversations. P5 reflected that he ‘Can't really express myself directly’ explaining that he likes his broad Yorkshire accent and that online he ‘can't talk proper’. The participants had differing explanations for this feeling of reduced expression. P2 found that communication was similar online to face to face but conveyed: ‘Almost the same, almost the same. Your half your body is oh erm, lower half I can't, I don't know but animated is easily, faces and names, yes it was almost naturally’. P7 also felt being online lacked ‘erm body language’ and rated online expression as nine out of ten and meeting in person as ‘Ten, ten’. P5 felt this related to living with aphasia: ‘No, almost the same, almost the same, in my my mind is mysterious ways. My mind is, it's trying to react naturally, trying to express myself naturally, the aphasia inhibits my expressions and the explanations’.

#### Subtheme 3: ‘Wetherspoons, Wetherspoons’: Loss of the physical environment

The participants expressed that despite enjoying interacting with the students online, they missed being in the same room. P3 wrote ‘Wetherspoons, Wetherspoons’ (a UK and Ireland‐based pub and hotel chain) when asked if he felt he would have got to know the students better if they were in the same room, indicating that he would have enjoyed meeting them in a pub. P7 gestured that being in the same room is ten out of ten.

The reasons for this preference varied between participants. P1 shared the importance of being with people, ‘But it's good because I come, I can speak to face to face and I can meet other people like me’ whilst P6 indicated he likes to get out for a drive to see people. P8 reflected that being in the same room was better for shy students, whereas for P3, students visiting him in his own home meant other people were there to provide support, ‘Yes, because I sometimes I was home with this and other people’. P5 felt that being together in person meant fewer interruptions than online, ‘Trouble is that the door knocking or ringing, oh dear, no’ although P8 shared that people were distracted by her cat at home.

## DISCUSSION

The aim of this study was to explore the experiences of PWA of taking part in an online conversation partner scheme during the COVID‐19 pandemic. Eight people were interviewed, and the data were analysed using reflexive thematic analysis, which resulted in two themes. These will be discussed here with reference to the existing literature, and then the implications will be considered.

### Theme 1: Communicating and connecting online: ‘It was brilliant’

The participants felt that the conversation partner scheme worked online between the PWA and the students. This fits with findings of studies exploring the perceptions of students and PWA of online conversation partner schemes, for example, Finch et al. (2020) whose student participants found the conversation partner scheme to be a positive experience and improved their confidence and proficiency in communicating with PWA, which was measured through ratings on a questionnaire. Furthermore, in a single group, pre‐test post‐test design using a verbal questionnaire, most of Lee et al.’s (2020) participants with aphasia found telepractice to be equally as suitable in terms of the psychosocial benefits from conversing with students. They highlighted the ease of interactions through telepractice as they could see the students up close. This evidence emphasizes the benefit of university‐run conversation partner schemes and the strength and skills of students as conversation partners for PWA.

Subtheme 1 demonstrated how the online conversation partner scheme had mutual benefits for both the PWA and the students. The students developed from being nervous at the start, to being helpful conversation partners, who could adapt to their partners’ communication and could facilitate supported conversations. For the PWA, it gave them a sense of purpose and fulfilment, knowing that they were helping others during the pandemic when they were feeling isolated, it also developed their own communication skills. These benefits of volunteering or supporting others have been reported in the wider aphasia literature. Brown et al. (2010) found that one of the key ways to live successfully with aphasia is ‘doing things’, including new activities, which can improve independence, a sense of achievement, purpose, usefulness, pleasure and well‐being, and can increase stimulation for the brain, and prevent boredom. Similarly. Grohn et al. (2014) found that engaging in activities related to the community, including volunteering, increased participation and a sense of enjoyment. Worrall et al. (2011) highlighted how PWA have goals to raise awareness of aphasia and to help train students.

The ‘mutual benefit’ of conversation partner schemes has also been highlighted in previous CPT studies. Cameron et al.’s (2018) participants with aphasia described in interviews and focus groups how training needs to be a ‘two‐way street’ (I've got to get something out of it. And so do they’. p.923), and found that there were benefits to themselves and others. Through semi‐structured interviews, students in Kearns and Cunningham's (2023):945 case study also described their conversation partner scheme as a ‘Mutually Beneficial Experience’. McMenamin et al.’s (2015):907 participants with aphasia discussed in interviews and focus groups how they were given the opportunity to help others within their (face‐to‐face) conversation partner programme. One participant explained: ‘It was good for me. … I feel proud if I help the students’. These positive findings also reflect those of studies that have shown changes to the language skills of PWA (Avent et al., 2009), and levels of competence and confidence in students (Finch, Cameron et al., 2017; Finch, Fleming et al., 2017; Nikkels et al., 2023). Shadden and Agan (2004) argue that one of the greatest challenges for PWA is to ‘renegotiate identity’. It seems that in the current study the conversation partner scheme gave the participants a sense of purpose and identity through having a meaningful role in teaching students.

Subthemes 2 and 3 showed how the participants in the current study found that they were still able to communicate, and to connect and build a relationship with the students, despite being online. Kearns and Cunningham (2023) also found that digital technology was a facilitator (as well as a barrier) for their participants. Neate et al. (2022) found benefits to communicating online, such as the fact that conversations were initiated because pictures and objects could be seen in people's backgrounds, and the participants’ own props could be used, helping them to feel in control of the interaction. Interestingly, their participants also spoke about being able to monitor themselves (e.g., their gestures) by watching themselves on Zoom. The participants in the current study who found the scheme to be successful acknowledged that this was influenced by personal factors. Menger et al. (2019) found that although aphasia can be a barrier to internet and technology use, this interacted with factors such as age, education and previous technology use. In this study, the participants ranged in age from their 40s to 80s, but irrespective of age and previous use of technology, they were all able to access the online format and were positive about communicating online.

The findings of these studies should be seen in the context of the COVID‐19 pandemic and the resulting social distancing measures. As Kong (2021) discusses, PWA faced further restrictions, in addition to their communication and physical difficulties, during the pandemic. Due to social distancing, they were not able to participate in community activities, which are critical for improving their quality of life in aphasia (Davidson et al., 2008), which for many will have increased the likelihood of social isolation and loneliness (Smith et al., 2020). Improving psychosocial well‐being through taking part in meaningful social activities, such as conversation partner schemes, became crucial (Bronken et al., 2012). Online platforms provided an opportunity for people to continue to have these meaningful interactions through social groups, conversation partner schemes, and speech and language therapy, and without the need to wear face masks.

In subtheme 4, the participants in this study described that they did not experience technology difficulties, they acknowledged the possibility and the need for help to resolve them. This reflects the findings of Nichol et al. (2023) who recognize the role of conversation partners in supporting PWA in access to technology. In favour of being online, the participants expressed how they found the ease and convenience of being online to be a benefit: They liked not having to leave their comfortable homes, where they could make themselves coffee, and were able to walk away if they were tired, in comparison to spending a long time travelling to ‘dreary’ rooms. Again, this mirrored findings by Neate et al. (2022), whose participants with aphasia found travel to be a challenge due to their aphasia and physical impairments, and also Kearns and Cunningham (2023) whose participants found the online format to be convenient at a time when public health restrictions were in place. This emphasizes the accessibility of online meetings for people who are not able to travel or do not live close to universities.

### Theme 2: Being me online: lacks ‘*Je ne sais quoi*’

Although the participants acknowledged benefits of the online conversation partner scheme, they felt that something was lacking, namely the human connection (subtheme 1). It could be the case that physical distance leads to emotional distance. Proximity to the therapist was a theme found by Lawton et al. (2018):1407 in their interview study on PWA's perceptions of the therapeutic alliance. Participants described close alliances being related to openness and ‘a human connection’ with the therapist and thus a relaxed atmosphere, with the therapeutic relationship going beyond professionalism. It could be the case that the participants in this study felt that this connection is more easily accessed when being in the same room together, as has been found in studies exploring therapeutic alliance in telehealth. In a study exploring social workers’ experiences of teletherapy, McCoyd et al.’s (2022):329 participants found it to be a ‘much more remote experience’, both physically and emotionally, despite the connection remaining strong. Their participants described the loss of subtle body language, which is usually useful in conveying ‘emotional indicators’. This difference to ‘connectedness’ could also be due to the formality, structure and the need to turn‐taking in online meetings, as well as the lack of mutual eye contact due to the use of webcams. Neate et al.’s (2022) participants found that turn‐taking on Zoom prevented informality, and that one‐to‐one conversations or asides could not happen when there were several people in a Zoom meeting.

Subtheme 2 provides insight into how the participants in this study also missed being able to fully express themselves and their personalities, including the use of all modes of communication. Given that PWA often use a range of modalities to communicate, including gesture, writing, drawing, facial expression, objects of reference and body language, being online restricted this. Kearns and Cunningham (2023):946 also found that the reduced options of communication ramps impacted the online conversation partner scheme and that student participants felt that ‘something was missing’. Neate's (2022):13 charity staff found it more difficult to interpret the communication and feelings of PWA without making use of all non‐verbal communication, and there was sometimes ‘missed communication’, when, for, example a raised hand was not seen.

Finally, subtheme 3 captures how the participants in this study felt that they missed being in different physical environments. As people who have had a stroke are often already limited in mobility, participation in conversation partner schemes, voluntary roles and community groups can, in normal circumstances, provide an opportunity to leave the house in a supported setting and to be in a range of physical environments, which can also mean avoiding the distractions of being at home and can support generalization of trained skills to different contexts.

## IMPLICATIONS

The findings of this study have implications for speech and language therapy courses, speech and language therapists and charities who run CPT and schemes. Participants in this study found that the conversation partner scheme worked online—they were still able to enjoy communicating and building a relationship with students. For many PWA who would benefit from taking part in a conversation partner scheme, but are unable to do this in person due to physical and/or geographical restrictions, having the opportunity to take part online could, as it did for participants in this study, develop their conversation skills, support their social participation, reduce isolation, and give them a sense of purpose. Even without restrictions, some still prefer to take part online due to the convenience and comfort of being able to stay in their own home. It is important to consider that ease of technological access and acceptability will depend on personal preferences, experience, skills and support, highlighting the importance of digital inclusion (Menger et al., 2016; Nichol et al., 2023).

Online conversation partner schemes should not be a substitute for face‐to‐face schemes for everyone, as there can be restrictions in communication, building a connection and self‐expression. Therefore, either a combined approach or being given the option to choose between the alternatives would be recommended. As Participant P2 put it, ‘I think it's a marriage between virtual and actual’. Interestingly, the participants talked about the importance of being in a range of locations which could support the idea of offering varied options for places to meet, for example, people's homes, universities, but also pubs, cafes, museums and libraries, as well as time spent together online. Meeting in person enables people to have conversations with each other but also to experience a range of interactions with people in society (Carroll et al., 2018).

## LIMITATIONS, CONTRIBUTIONS AND FUTURE DIRECTIONS

There are limitations which need to be recognized in this study. The relatively small sample size included eight participants with aphasia. However, it is acknowledged that qualitative research often involves small sample sizes and that, through semi‐structured interviews, researchers gather rich data, detailing in depth accounts of the participants’ experiences (Willig, 2022). The participants were motivated to participate, which may reflect their positive experience of the scheme.

This study followed the COnsolidated criteria for REporting Qualitative research checklist (COREQ, Tong et al., 2007). There are two items which could not be completed for this study. It has not been possible to carry out participant checking as not all participants were contactable post‐study and not all participants would have been able to read the transcripts. With regards to describing the sample, the demographic data of the participants, including their aphasia diagnosis, was limited. The eight participants were referred to the CPT scheme and had been accepted as eligible to take part as they had post stroke aphasia, could engage in supported conversations, and did not present with any significant cognitive impairment. Specific information on their aphasia diagnosis was not recorded but, in the interviews, it was evident that they presented with a range of mild–severe expressive difficulties and mild–moderate receptive difficulties. Particularly given COVID‐19, information on their home environment, social support networks and general well‐being may have provided insight into their experience of lockdown, which may have impacted on their perceptions of the CPT.

During the write up of this study, other related studies have been published. Most of these differed in designs and methods, for example, had quantitative or mixed‐methods designs (Finch et al., 2020; Lee et al., 2020; Power et al, 2022). Kearns and Cunningham (2023) and Power et al. (2020) had a similar design and focus: they carried out semi‐structured interviews of PWA who took part in a similar CPT scheme for students during COVID‐19, although they also collected survey data from students. Some of the findings of the current study complement the results from Kearns and Cunningham (2023), such as acceptability and perceived convenience of the online CPT, the opportunities for communicating and maintaining social connections in a global pandemic, and the mutual benefit to PWA and students. This study provides concurrent evidence that an online CPT is acceptable in a UK‐based university. Novel contributions of this study were findings related to PWA missing the human connection, the restriction on self‐expression and the loss of the physical environment experienced by participants.

Future research questions could include exploring the ongoing acceptability of online conversation partner schemes post COVID‐19. It was recognized that during COVID‐19, social isolation and boredom were heightened, and it is important to understand if post COVID‐19 there is the same need and acceptability of online conversation partner schemes. Additionally, there should be an evaluation of post‐COVID‐19 schemes which combine face‐to‐face and online sessions, which the participants felt would be the optimal format. The perspectives of both students and PWA should be explored as these are vital to the success of these schemes. Considering some participants found using a range of communication modalities difficult online, research that focuses on developing videoconferencing platforms that are designed around the needs of PWA could be beneficial (Neate et al., 2022). The researchers are also interested in exploring further how PWA feel they express themselves and the impact of their aphasia on this self‐expression, both in person and online. Finally, it would be useful to explore how the ability to engage with CPT and telepractice more generally relates to aphasia presentation, severity and self‐efficacy.

## CONCLUSIONS

Exploring the experiences of PWA who took part in an online conversation partner scheme during COVID‐19 has shown that from their perspective the scheme is acceptable online and has benefits for both students and PWA. However, for some, being online does not provide quite the same experience as interacting and building a rapport with someone in person. Having the option of meeting online or in person will create opportunities for PWA to take part in CPT schemes, which can reduce isolation, give a sense of purpose and improve participation. By sharing important insights from people with a range of types and severities of aphasia, this study has contributed to the existing literature and has provided implications for those providing CPT. This work supports the field's understanding on how we can continue to expand on the way we offer accessible services to PWA and their social networks.

## CONFLICT OF INTEREST STATEMENT

The authors report no conflict of interest.

## Data Availability

The data that support the findings of this study are available from the corresponding author upon reasonable request.
